# Beyond traditional translation: ncRNA derived peptides as modulators of tumor behaviors

**DOI:** 10.1186/s12929-024-01047-0

**Published:** 2024-06-14

**Authors:** Kang Wen, Xin Chen, Jingyao Gu, Zhenyao Chen, Zhaoxia Wang

**Affiliations:** 1 Cancer Medical Center, The Second Affiliated Hospital of Nanjing Medical University, Nanjing, Jiangsu 210011 P.R. China; 2 Department of Respiratory Endoscopy, Shanghai Chest Hospital, Shanghai Jiao Tong University School of Medicine, Shanghai, 200030 P.R. China; 3 Department of Oncology, Shanghai Medical College, Fudan University, Shanghai, 200032 China

**Keywords:** ncRNA, Protein, Peptides, circRNAs, lncRNAs, Cancer

## Abstract

Within the intricate tapestry of molecular research, noncoding RNAs (ncRNAs) were historically overshadowed by a pervasive presumption of their inability to encode proteins or peptides. However, groundbreaking revelations have challenged this notion, unveiling select ncRNAs that surprisingly encode peptides specifically those nearing a succinct 100 amino acids. At the forefront of this epiphany stand lncRNAs and circRNAs, distinctively characterized by their embedded small open reading frames (sORFs). Increasing evidence has revealed different functions and mechanisms of peptides/proteins encoded by ncRNAs in cancer, including promotion or inhibition of cancer cell proliferation, cellular metabolism (glucose metabolism and lipid metabolism), and promotion or concerted metastasis of cancer cells. The discoveries not only accentuate the depth of ncRNA functionality but also open novel avenues for oncological research and therapeutic innovations. The main difficulties in the study of these ncRNA-derived peptides hinge crucially on precise peptide detection and sORFs identification. Here, we illuminate cutting-edge methodologies, essential instrumentation, and dedicated databases tailored for unearthing sORFs and peptides. In addition, we also conclude the potential of clinical applications in cancer therapy.

## Introduction

According to previous studies, about 98% of the tens of thousands of mammalian transcripts are noncoding RNA (ncRNA) [1–6]. These ncRNAs skip protein translation as they are transcribed from the genome [7]. Nevertheless, they serve a multitude of roles at varying stages and in diverse regions, including transcription regulation, chromosomal modification, epigenetic alterations, protein modification, and medical resistance, among others [5, 8–15]. Certain ncRNAs have even been employed as clinical diagnostic and prognostic markers [16–27]. Intriguingly, a growing body of research has discovered that ncRNAs, originally deemed incapable of encoding proteins and peptides, can synthesize bioactive peptides [28–38]. Small open reading frames (sORFs) within ncRNAs, which typically span less than 300 nucleotides and produce peptides of less than 100 amino acids, are responsible for encoding these peptides [39–48]. Owing to recent advances in detection technology, the capacity to identify peptides and sORFs has substantially improved, unmasking previously undetectable peptides and shedding light on their vital biological functions. Predominantly, these peptides are encoded by long noncoding RNAs (lncRNAs), circular RNAs (circRNAs), and primary microRNAs (pri-miRNAs).

Traditional open reading frames (ORFs), which span over 300 nucleotides and have a canonical start codon (AUG), are larger than ncRNA sORFs, which are smaller. This divergence is a key factor in the underappreciation of ncRNAs' capacity for encoding [49, 50]. However, recent advancements in multi-omics have rendered the identification, validation, and functional characterization of sORFs and peptides achievable. Researchers have improved mass spectrometry (MS) methods to more precisely detect ncRNA sORFs and their encoded peptides. By employing more accurate ribosome analysis, they can now differentiate between genuine functional peptides and other peptides, thereby better revealing the encoding potential and role of sORFs [49, 51–56]. Generally, ribosome analysis offers a genome-wide snapshot of actively translated regions within a cell [57–60]. Additionally, as the ribosome scans sequences one codon at a time, the information read maintains a precise three-nucleotide cycle and exhibits single-nucleotide resolution [61]. Nonetheless, sORFs identification remains challenging. Currently, in addition to ribosome sequencing (Ribo-seq), sORFs can be predicted through bioinformatics tools, such as sORFs Finder [62].

ncRNAs classified as lncRNAs by researchers are those that have more than 200 nucleotides and perform important roles in living things, particularly in the context of malignancies [6, 63–71]. Existing studies have discovered that lncRNAs possess small open reading frames (sORFs) capable of encoding peptides, with most peptides originating from lncRNAs. Regulatory elements upstream of ORF, such as internal ribosome entry sites (IRES), have been found to mediate the translation of peptides [72, 73]. IRES elements are primarily located in the 5 'untranslation region upstream of the ORF controlled by IRES. They facilitate sORFs regulatory RNA sequence transfer by recruiting ribosomes and conducting ribosome assembly without relying on the 5' cap structure. Additionally, IRES elements may reside between and within ORFs to mediate translation, subsequently translating the continuous sORFs of lncRNAs into peptides [74–78]. m6A modification is common in mammals, primarily serving to modify and regulate mammalian gene expression, as well as RNA stability, localization, splicing, and translation at the post-transcriptional level [79–88]. Recent advancements in ribosome analysis, computational prediction, and mass spectrometry have revealed that m6A-driven endogenous ncRNA translation is widespread. m6A reading protein YTHD3 closely binds to translation initiation factor eIF4G2, which promotes intracellular circRNA translation [89]. The m6A sites on lncRNA were experimentally verified and then analyzed by mutating the m6A sites that determine the translation of small peptides by lncRNA. A cell model was constructed by CRISPR/Cas9 method to study the function of small peptides encoded by lncRNA in tumor cells. Finally, it was found that lncRNA AFAP1-AS1 translation axis suggests that N6-methyladenosine in its 5 '-UTR is a node-regulator of peptide translation [32, 90, 91].

CircRNA is a kind of atypical reverse splicing ncRNA [92–94]. Since its first discovery in the virus, circRNA has been considered as abnormal transcriptional noise [95–97]. However, advancements in bioinformatics and second-generation sequencing have unveiled the roles of circRNA [98–106]. CircRNA can act as a miRNA sponge, regulating the miRNA-mRNA axis, as a transcriptional regulator, and as a prognostic marker [107–127]. Formed by reverse splicing, circRNA lack the 5 'cap and 3' end, which makes them resistant to ribonuclease and therefore are thought to lack the traditional translation promoter [128–131]. Numerous recent studies have discovered that circRNA contains sORFs, which also possess peptide-encoding functions [32, 132]. In addition to peptide translation mediated by IRES elements and m6A, two peptide translations similar to lncRNA, there is also an endogenous rolling translation process that terminates by the codons outside the ring. This unique translation mechanism contributes to the complex and multifaceted role circRNA plays in biological processes [89, 129, 133–142].

As a precursor to microRNA (miRNA), pri-miRNA contain hairpin, 5 '-cap, and 3' -polyadenylate (AAA) sequences that share the same characteristics as other mRNA [143]. Now pri-miRNA has been found to contain sORFs, which can encode plant-related peptides and have important applications in agriculture [144].

NcRNA-encoded peptides have been demonstrated to participate in diverse physiological and pathological processes within the human body, encompassing embryonic development, muscle formation and regeneration, metabolism, stress responses, inflammation, and immune regulation [32, 145–151]. Crucially, these peptides also hold significant sway over tumorigenesis and progression, influencing aspects such as tumorigenesis, proliferation, invasion, and metastasis [152–155]. In the subsequent discussion, we will show the different come of peptides and inhibiting cancer and elucidate their respective mechanisms, further highlighting the intricate and multifaceted roles these peptides play in biological processes.

### Peptides encoded by ncRNA

#### Peptides from lncRNA

Long noncoding RNAs (lncRNAs), one of the most common types of ncRNA, were among the first to garner researchers' attention for their peptide-encoding potential [156]. Recent research exploring this possibility uses a variety of methods, including ribosome profiling, MS-based proteomics, microscopy, and CRISPR-based genetic screening. This approach has uncovered hundreds of non-canonical lncRNA coding DNA sequences (CDSs) capable of producing stable functional peptides essential for cell growth. Consequently, peptides encoded by lncRNAs are now less likely to be considered "translation noise" and more as functional peptides [157]. For instance, LncFORCP, predominantly found in the cytoplasm of normal colon and stomach cells, is regulated by the transcription factor FOXA1. This lncRNA encodes a 79-amino acid peptide named FORCP, largely localized in the endoplasmic reticulum (ER) [158]. Another lncRNA, AC025154.2, has the potential to code an acid peptide, MIAC, which acts as an inhibitor of the actin cytoskeleton [159, 160]. Furthermore, LINC00665, mainly located in the cytoplasm and possessing four open reading frames (ORFs), has one ORF (ORF1) encoding the peptide CIP2A-binding peptide (CIP2A-BP) [161].

#### Peptides from circRNA

CircRNA, as the first known ncRNA capable of encoding peptides, wields a pivotal role in biological systems, owing to its unique structure and distinctive translation process [162–164]. CircMAPK1, primarily located in the cytoplasm, encompasses an open reading frame (ORF) initiated by the start codon ATG and guided by an internal ribosome entry site (IRES). It gives rise to a 109-amino acid peptide, MAPK1-109 aa, known for its tumor proliferation inhibitory properties [165]. Hsa_circ_0061137, or CircDIDO1, originates from the back splicing of exons 2–6 of the linear DIDO1 gene transcript. Spanning 1787 nucleotides in length, it is predominantly found in the nucleus and cytoplasm of gastric cancer (GC) cells. CircDIDO1 exhibits a marked downregulation in GC cells compared to normal gastric mucosal epithelial cells [166]. Similarly, both circSEMA4B and its encoded peptide, SEMA4B-211aa, manifest at lower levels in breast cancer (BC) and function as tumor suppressors both in vivo and in vitro [167]. Lastly, hsa-circ-0000437, featuring a 144nt ORF that encodes the peptide CORO1C-47aa through IRES, adds to the complexity and variety of the circRNA-peptide landscape [168].

#### Peptides from other ncRNAs

Advancements in research have unveiled that beyond lncRNA and circRNA, there exist other particular categories of ncRNAs that also encode peptides. One such example is MiPEP133, a microprotein composed of 133 amino acids, encoded by the open reading frame (ORF) within the primary miRNA of miR-34a. The expression of MiPEP133 is especially elevated in several human tissues including the colon, ovaries, stomach, uterus, and pharynx. Predominantly located within the mitochondria, its expression within the cytoplasm remains relatively low [169]. When miPEP-8 is overexpressed or knocked down, it affects Drosophila development. Combining genetic and molecular approaches and genome-wide transcriptome analysis, we found that miR-8 expression is independent of miPEP-8 activity, and that miPEP-8 and miR-8 regulate the expression of hundreds of genes in parallel [170]. Peptide encoded by pri-miRNA-31 suppresses autoimmunity by promoting Treg differentiation [171]. The polypeptides produced by pri-miR171b in Arabidopsis and pri-miR165a in Arabidopsis (which we refer to as miPEP171b and miPEP165a, respectively) enhance the accumulation of the corresponding mature miRNAs, which leads to the down-regulation of target genes involved in root development [144].

Regardless of which ncRNA encode peptides, more and more research confirm that these peptides play an important role in living organisms, especially in tumor (Tables 1 and 2).

**Table 1:** Tumor suppressing effects

Phenotype	Category	Peptide	Cancer	Function
Tumor suppressing effects	circRNA	MAPK1-109aa	Gastric cancer	Inhibits the progression of gastric cancer by inhibiting the activation of MAPK signaling
	lncRNA	FORCP	Colorectal cancer	Regulates apoptosis and tumorigenicity in well-differentiated CRC cells
	lncRNA	TP53LC04	Cancer	Inhibits cell proliferation by regulating the cell cycle in response to DNA damage
	lncRNA	MIAC	Head and neck squamous cell carcinoma	Regulates SEPT2 (Septin 2)/ITGB4 (integrin Beta 4) to directly interact with AQP2 (Aquaporin 2), thereby inhibiting actin cytoskeleton and ultimately inhibiting tumor growth and metastasis in HNSCC
	pri-miRNA	miPEP133	Cancer	Interacts with mitochondrial heat shock protein 70kD (HSPA9) and prevents HSPA9 from interacting with its binding partner, resulting in a decrease in mitochondrial membrane potential and mitochondrial mass
	lncRNA	CIP2A-BP	Triple-negative breast cancer	Inhibition of PI3K/AKT/NF-κB pathway by CIP2A-BP micropeptide leads to decreased expression of MMP-2, MMP-9, and Snail, which significantly reduces lung metastasis and improves overall survival
	circRNA	DIDO1-529aa	Gastric cancer	It directly interacts with poly ADP-ribose polymerase 1 (PARP1), specifically binds peroxidase-reducing protein 2 (PRDX2), and promotes RBX1-mediated ubiquitination and degradation of PRDX2
	circRNA	CORO1C-47aa	Endometrium tumor	Plays a negative regulatory role in endometrial tumor angiogenesis
	lncRNA	ASRPS	Triple-negative breast cancer	Binds STAT3 and downregulates STAT3 phosphorylation, resulting in decreased VEGF expression
	lncRNA	HBVPTPAP	Hepatocellular carcinoma	The apoptosis of hepatocellular carcinoma cells was induced by regulating JAK/STAT signaling pathway
	lncRNA	PEP-AP	Colorectal cancer	Attenuated the pentose phosphate pathway and sensitized colorectal cancer cells to oxaliplatin
	lncRNA	KRASIM	Hepatocellular carcinoma	Decreased KRAS protein levels, resulting in inhibition of ERK signaling activity in HCC cells
	long intergenic noncoding RNAs (lincRNAs)	MP31	Glioblastoma	Restriction of lactate-pyruvate conversion in mitochondria by competition with mitochondrial lactate dehydrogenase (mLDH)
	circRNA	SEMA4B-211aa	Breast Cancer	Inhibits the production of PIP3 and thus the phosphorylation of AKT (Thr308)

**Table 2:** Tumor promoting effects

Phenotype	Category	Peptide	Cancer	Function
Tumor promoting effects	lncRNA	ASAP	Colorectal cancer	Enhanced ATP synthase construction, increased ATP synthase activity and mitochondrial oxygen consumption rate, promoting the proliferation of colorectal cancer cells
	circRNA	AXIN1-295aa	Gastric cancer	Activation of Wnt/β-catenin signaling pathway promotes gastric cancer progression
	circRNA	circHNRNPU_603aa	Multiple myeloma	The proliferation of MM cells was promoted
	circRNA	circCHEK1_246aa	Multiple myeloma	Causes chromosomal instability and induces bone degeneration in multiple myeloma
	lncRNA	Linc013026-68AA	Hepatocellular carcinoma	In some HCC cells and plays a role in cell proliferation
	circRNA	Hsa_circ_0006401	Colorectal cancer	Promotes the stability of host gene COL6A3 mRNA, thereby promoting CRC proliferation and metastasis
	circRNA	EIF6-224aa	Triple-negative breast cancer	TNBC progression was promoted by stabilizing MYH9 and activating Wnt/β-catenin pathway
	lncRNA	sPEP1	Neuroblastoma	Inhibited serum deprivation induced senescence and promoted spherogenesis, growth or metastasis of NB stem cells
	lncRNA	MPM	Hepatocellular carcinoma	Promote hepatocellular carcinoma metastasis by enhancing mitochondrial complex I activity
	circRNA	circCOL6A3_030_198aa	Gastric cancer	Functions as a tumor promoting factor
	circRNA	cGGNBP2-184aa	Intrahepatic cholangiocarcinoma	Directly interacts with STAT3, phosphorylates STAT3Tyr705, and plays a positive regulatory role in regulating IL-6/STAT3 signaling
	circRNA	circMRPS35-168aa	Hepatocellular carcinoma	Promotes cisplatin resistance in HCC
	lncRNA	PACMP	Cancer	Regulate cancer progression and drug resistance by modulating DDR
	lncRNA	XBP1SBM	Triple-negative breast cancer	The XBP1s pathway promotes TNBC angiogenesis and metastasis
	lncRNA	APPLE	Hematopoietic malignancy	Promotes PABPC1-EIF4G interaction and promotes mRNA cyclization and eIF4F initiation complex assembly to support specific cancer-promoting translation programs

### Functions and Mechanisms of ncRNA-Encoded Peptides/Proteins in Cancers

#### Influence on cancer proliferation

In the process of tumor formation and development, different phenotypes will appear at different stages, among which the most common phenotype is the uncontrolled proliferation of tumor cells. It is now confirmed that the peptides encoded by ncRNA can play an inhibitory role in the process of tumor proliferation. MAPK1-109 aa is a 109-amino acid peptide encoded by circMAPK1 in the cytoplasm via sORFs containing IRES. The researchers found that the peptide could reduce the phosphorylation level of MAPK1 and inhibit MAPK1 and its downstream factors by competing with MEK1, thus inhibiting tumor proliferation [165]. The proliferation of highly differentiated colorectal cancer has always been a focus of research. LncFORCP, an ncRNA regulated by the transcription factor FOXA1, encodes the 79-amino acid FORCP peptide. Further studies have shown that FORCP can promote the proliferation and tumorogenesis of highly differentiated colorectal cancer cells under endoplasmic reticulum stress by inhibiting the function of BRI3BP protein, and inducing cancer cell apoptosis, which is expected to become a new therapeutic target [158]. The birth of bioinformatics has greatly increased the ability of researchers to predict and screen ncRNA. Using bioinformatics and CRISPR-Cas9 technology, we screened hundreds of lncRNA with coding potential in HepG2 cells. TP53LC04 was the most obvious. Further experiments demonstrated that the peptide TP53LC04 induced by tp53 could inhibit the proliferation and regulate the cell cycle of human cancer cells, but did not affect its lncRNA, suggesting that this peptide may play an important role in the tumor inhibition regulated by tp53, especially in the DNA damage response [172]. MIAC is a peptide that inhibits actin cytoskeleton and is encoded by lncRNA AC025154.2. Overexpression of MIAC significantly inhibited tumor growth in head and neck squamous cell carcinoma (HNSCC) and produced fewer lung metastatic nodules. Experiments have shown that when MIAC directly interacts with aquaporin 2(AQP2). MIAC directly binds to the Y221, L217 and E232 amino acid sites on AQP2 protein to play a biological role. AQP2 regulates Integrin Beta 4(ITGB4) and Septin 2(SEPT2), ultimately regulating SEPT2/ITGB4 signaling, affecting actin cytoskeleton, inhibiting proliferation and ultimately achieving antitumor effects [159, 160]. Ribosome analysis of HuH-7 cells, followed by RiboCode analysis of the HuH7 Ribo-seq dataset, revealed that lncRNA NCBP2-AS2 had a highly preserved novel translation. KRASIM specifically binds to KRAS protein, and overexpression of KRASIM reduces the level of KRAS protein, leading to inhibition of ERK signaling pathway activity in hepatoma cells. Finally, it was found that KRASIM overexpression inhibited HCC cell proliferation by inhibiting the KRAS/ERK pathway [173].

**Fig. 1:**
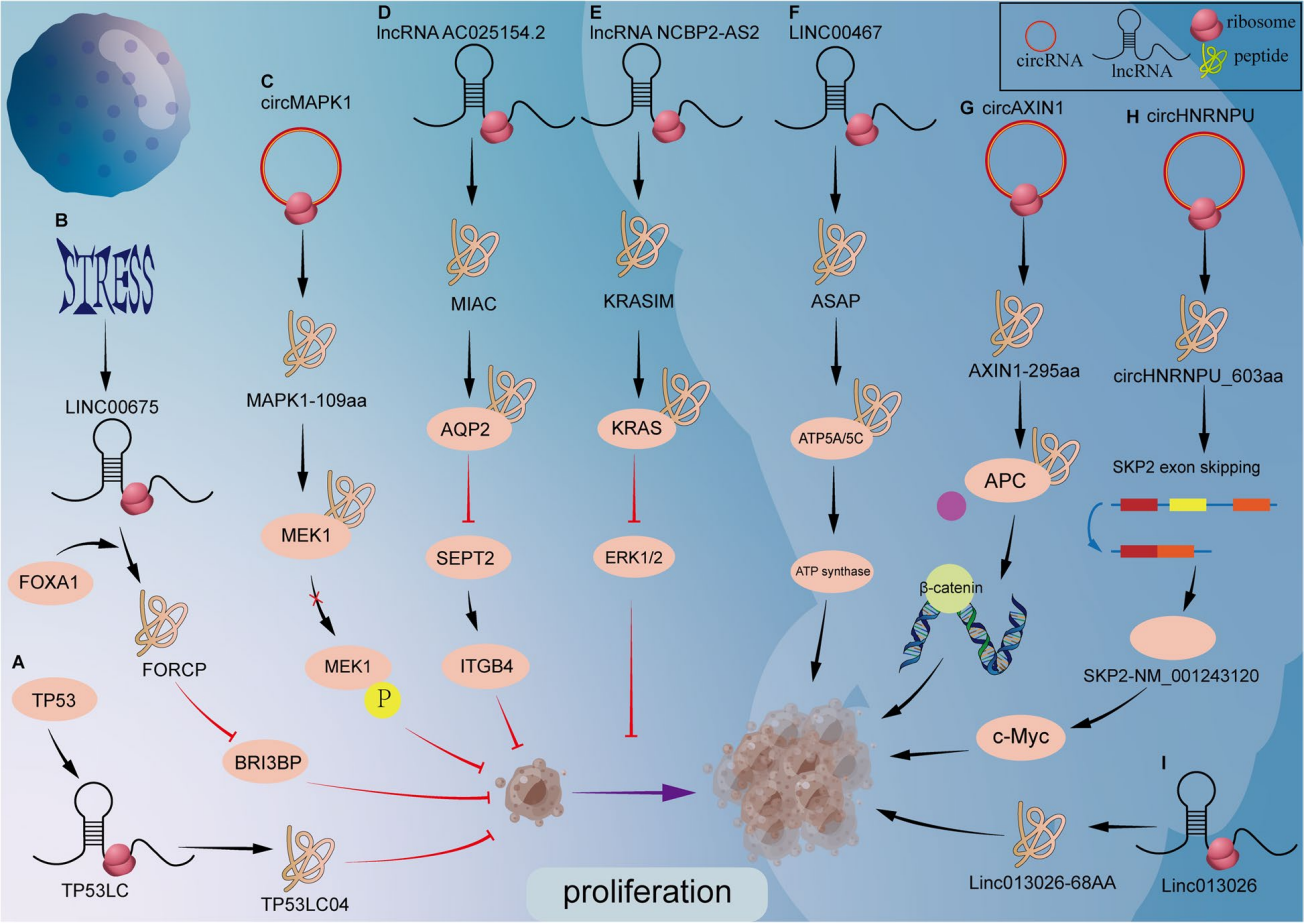
The mechanism of peptides in the process of tumor proliferation. **A** TP53-induced peptide TP53LC04 inhibited the proliferation. **B** FORCP peptide inhibited basal cell proliferation and induced cell apoptosis by inhibiting BRI3BP protein function in well-differentiated colorectal cancer cells under ER stress. **C** MAPK1–109aa inhibited MAPK1 phosphorylation through competitive binding, and inhibiting the activation of MAPK1 and its downstream factors in the MAPK pathway. **D** MIAC interacts with AQP2 (Aquaporin 2) to inhibit the actin cytoskeleton by regulating SEPT2 (Septin 2)/ITGB4 (Integrin Beta 4) and ultimately suppressing the tumor growth and metastasis of HNSCC. **E** KRASIM decreases the KRAS, leading to the inhibition of ERK signaling activity. **F** ASAP enhanced the ATP synthase construction by interacting with the subunits α and γ (ATP5A and ATP5C), increasing ATP synthase activity and mitochondrial oxygen consumption rate. **G** CircAXIN1 encodes AXIN1-295aa, which competitively binds to APC, leading to the release and nuclear translocation of β-catenin. **H** MM cells secrete circHNRNPU into the BM microenvironment to regulate SKP2 exon skipping and inhibit c-Myc ubiquitin. **I** Linc013026-68AA is expressed in HCC cells and plays a role in cell proliferation

Some peptides inhibit tumor cell proliferation, and naturally, there are peptides encoded by ncRNA that promote tumor cell proliferation. The ORF1 of LINC00467 encodes a small 94-amino acid peptide ASAP, which is associated with the inner mitochondrial membrane. It was confirmed that ASAP formed a complex with ATP5A and ATP5C, and D65 residues of ASAP increased ATP synthase activity and mitochondrial oxygen consumption rate by enhancing the interaction between ATP5A and ATP5C. Finally, ASAP promotes tumor growth by regulating mitochondrial ATP [174]. The Wnt/β-catenin signaling pathway is a classic signaling pathway. Researchers have recently found that CircAXIN1 encodes a 295-amino acid peptide AXIN1295aa that interacts competitively with APC to occupy AXIN1's position in the destruction complex. Thus, the Wnt/β-catenin signaling pathway is activated to promote the occurrence and development of GC and enhance the proliferation of GC cells [175]. circHNRNPU_603aa encoded by circHNRNPU could significantly improve the growth rate of tumor cells, while cell proliferation was significantly inhibited after circHNRNPU_603aa was silenced. circHNRNPU-603aa up-regulates the splicing isomers of circHNRNPU603aa and SKP2-NM_001243120 by mediating SKP2 selective splicing. Thus, circHNRNPU-603aa competitively inhibits c-Myc ubiquitination [176]. Linc013026-68AA contains a peptide segment of 68 amino acids, which is mainly located in the perinuclear region after Myc specific staining. Researchers have demonstrated that Linc01302668AA overexpressed HeLa cells have a growth rate approximately 1.7 times higher than that of control vector transfection. Reduction of Linc013026-68AA in HepG2 cells resulted in a reduction in the cell proliferation rate of about 2 times that of control vector transfection within 3 days [177] (Fig. 1).

### Influence on *cancer* metastasis

Another characteristic of tumors is the wide range of metastasis and spread, and it has been found that ncRNA-encoded peptides can effectively inhibit this phenotype. miPEP133, a microprotein encoded by ORF in pri-miRNA of miR-34a, is highly expressed in the human colon, ovary, stomach, uterus, and pharynx tissues. MiPEP133 binds HSPA9 in mitochondria, prevents HSPA9 from interacting with HSP60, TIM44, and VDAC1, and inhibits the normal function of HSPA9 as a mitochondrial companion, eventually leading to mitochondrial mass loss, reduced mitochondrial membrane potential and ATP production [169].In the ncRNA study for breast cancer, LINC00665 has four sORFs, in which ORF1 encodes the CIP2A binding peptide CIP2A-BP. It binds to CIP2A, inhibits PI3K/AKT/NFjB pathway and downstream target protein (MMP2, MMP9, and Snail) expression through CIP2A-mediated PP2A, and inhibits TNBC migration and invasion. CIP2A-BP can be used as a potential therapeutic target to effectively inhibit breast cancer metastasis through inhibition of the PI3K/AKT/NFjB pathway. New mice MMTV-PyMT that can reduce lung metastasis were generated by introducing CIP2A-BP into C57BL/6 mice and then mating with MMTV-PyMT mice; CIP2A-BP + / + . To further investigate the metastasis inhibitory effect of CIP2A-BP, injection of CIP2A-BP through the mammary pad significantly reduced the number of lung metastatic sites, as well as significantly reduced the p-AKT level of the primary tumor. In addition, CIP2A-BP injection through the tail vein revealed that mice injected with CIP2A-BP had a significantly higher survival rate and fewer lung metastatic sites compared with control mice. These results suggest that CIP2A-BP can effectively inhibit the metastasis and invasion of breast cancer, thus improving the overall survival rate [161].CircDIDO1 is mainly distributed in the nucleus and cytoplasm of gastric cancer (GC) cells. CircDIDO1 has IRES, ORF, and m6A modifications, and is capable of encoding the peptide DIDO1-529aa. DIDO1-529aa could interacted with both 1–372 aa and 525–1014 aa domains of PARP1 protein, which not only inhibits the binding of PARP1 to damaged DNA and the enzymatic activity of PARP1, but also increased levels of cleaved caspase 3 and cleaved PARP1 in GC cells, and finally inhibits the apoptosis and transfer of GC cells [166]. Micropeptide in Mitochondria (MPM) is significantly down-regulated in human HCC tissues and inhibits mitochondrial complex I activity, mitochondrial respiration, and ATP production. When MPM is highly expressed, MPM works with NDUFA7 to inhibit the level of NAD + /NADH, thereby inhibiting the metastasis of tumor cells. Further studies showed that miR-17-5p could directly bind to mRNA 3 '-UTR to inhibit the expression of MPM, and the up-regulation of miR-17-5p was significantly correlated with the down-regulation of MPM in HCC tissues [178].CircSEMA4B and peptide SEMA4B-211aa were expressed at low levels in breast cancer(BC) and exerted as tumor suppressors in vivo and in vitro. Co-IP assay proved that SEMA4B-211aa inhibits the formation of the p85/p110 complex by forming a complex with free p85. This results in decreased p110 protein and decreased PI3K signal. The reduction of the p85/p110 complex inhibits the production of PIP3 and thus the phosphorylation of AKT (Thr308). Finally, the metastasis of BC was inhibited [167].

**Fig. 2:**
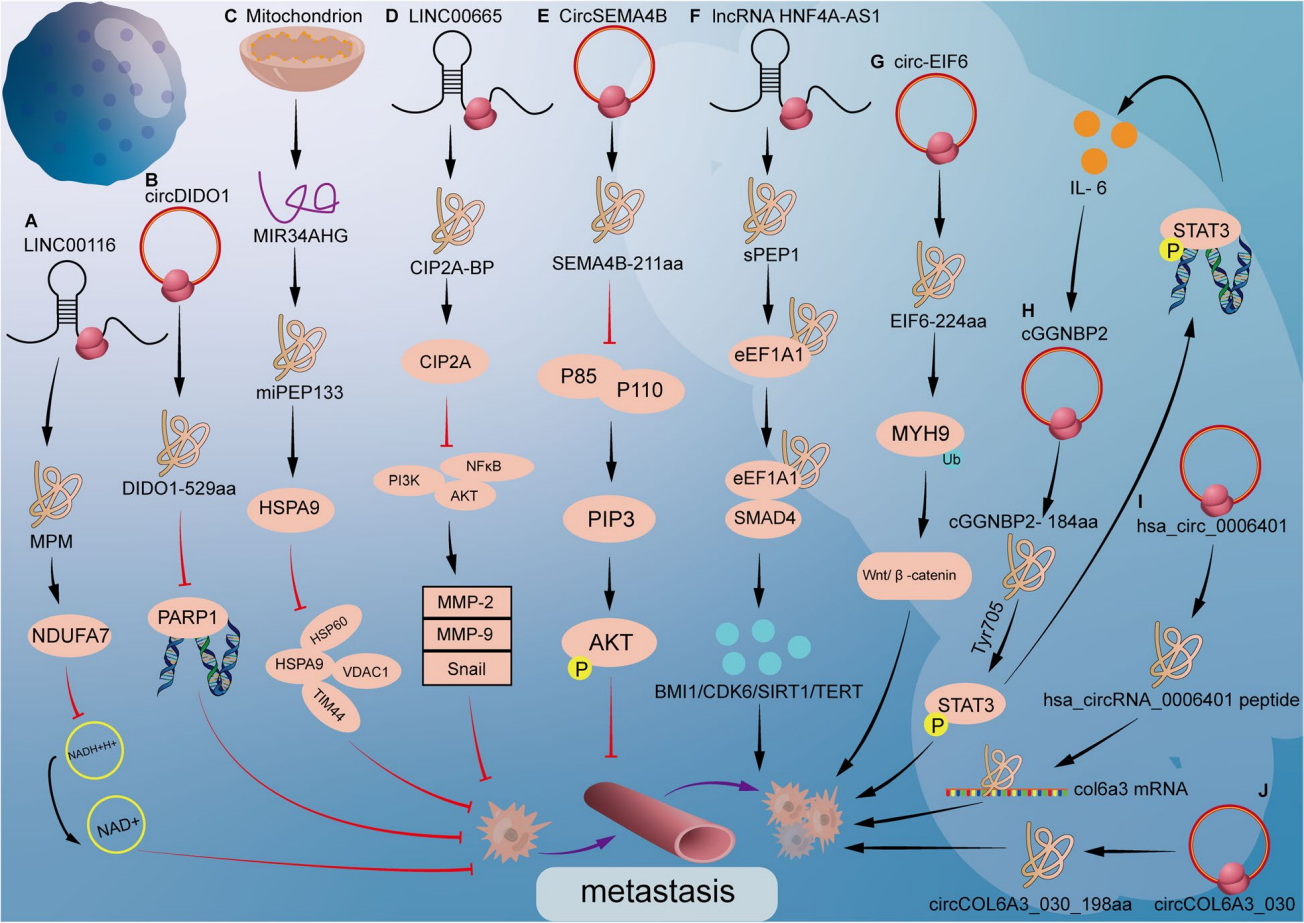
The mechanism of peptides in the process of tumor metastasis. **A** MPM acted with NDUFA7 to inhibit the level of NAD + /NADH, thus inhibiting the metastasis of tumor cells. **B** circDIDO1 encoded a novel 529aa protein that directly interacted with poly ADP-ribose polymerase 1 (PARP1) and inhibited its activity. **C** miPEP133 prevent HSPA9 from interacting with its binding partners, leading to the decrease of mitochondrial membrane potential and mitochondrial mass. **D** CIP2A-BP binds CIP2A, thus releasing PP2A activity, resulting in decreased expression levels of MMP-2, MMP-9, and Snail. **E** SEMA4B-211aa inhibits the formation of p85/p110 complex then decreased p110 protein and decreased PI3K signal, finally inhibiting the metastasis. **F** sPEP1 directly binds to eEF1A1 to promote its interaction with SMAD4, upregulation of downstream target genes, and promotion of self-renewal and tumor metastasis. **G** EIF6-224aa directly interacted with MYH9, and decreased MYH9 degradation by inhibiting the ubiquitin–proteasome pathway and subsequently activating the Wnt/β-catenin pathway. **H** cGGNBP2- 184aa interacts with the STAT3, phosphorylates its Thy705 site and initiates the transcription of downstream target genes of STAT3. **I** Hsa_circ_0006401 peptides decreased the mRNA and protein level of the host gene col6a3 by promoting col6a3 mRNA stabilization. **J** circCOL6A3_030 promoted GC cell migration by encoding a small peptide called circCOL6A3_030_198aa

And then some peptides promote tumor metastasis. Hsa_circ_0006401-198aa is encoded by Hsa_circ_0006401, which regulates the growth, migration, and metastasis of colorectal cancer. By means of immunoprecipitation-combined mass spectrometry, gene body analysis, and mRNA decay analysis, hsa_circ_0006401-198aa promoted the stability of host gene COL6A3 mRNA, thus promoting the metastasis of colorectal cancer [179]. circ-EIF6 (hsa_circ_0060055) encodes a 224-aa peptide (EIF6-224aa) in a study to determine the role of the Wnt/ β-catenin pathway in tumor metastasis. EIF6-224aa can up-regulate target genes in the Wnt/ β-catenin pathway, and it interacts with MYH9 to activate the Wnt/ β-catenin pathway and further promote TNBC cell metastasis [180]. HNF4A-AS1 encodes a small 51-amino acid peptide called sPEP1, which directly interacts with eukaryotic translation extension factor 1α-1 (eEF1A1) to promote its binding to SMAD family member 4 (SMAD4) and subsequently upregulates stem cell genes associated with tumor progression and promotes tumor metastasis [181]. Experiments showed that cGGNBP2-184aa directly interacts with signal transducers and activators of transduction3 (STAT3) to phosphorylate STAT3. This peptide plays a positive regulatory role in IL-6 /STAT3 signaling, forming a positive feedback loop signaling pathway. Finally activate the JAK-STAT signal and promote cell metastasis [182]. circCOL6A3_030 promoted GC cell migration by encoding a small peptide called circCOL6A3_030_198aa [183] (Fig. 2).

### Influence on *cancer* angiogenesis

Angiogenesis, not only allows tumor cells to metastasize rapidly in a large range but also provides nutrients for tumor cells to meet their proliferation needs. Similarly, peptides encoded by ncRNA have been found to play an important role in promoting tumor angiogenesis. hsa-circ-0000437 has a 144nt ORF that encodes a peptide called CORO1C-47aa via IRES. CORO1C-47aa binds to transforming acidic coiled coil 3 (TACC3) through its PAS-B domain to compete with ARNT to inhibit VEGF expression, while TACC3 promotes the recruitment of ARNT protein to the HRE site and promotes VEGF gene transcription. In conclusion, CORO1C-47aa can promote endothelial cell proliferation and migration by up-regulating and down-regulating VEGFA and VEGFR2 protein expression [168]. The ORF of LINC00908 encodes 60-aa peptide (6.62KD) ASRPS. Through the detection of TNBC tissue samples, it was found that ASRPS was negatively correlated with p-STAT3 expression. Experiments have confirmed that ASRPS directly bind to STAT3, inhibit STAT3 phosphorylation and its transcriptional activity, and regulate the expression of VEGF through STAT3 phosphorylation. In the mouse xenograft model, the expression of ASRPS was negatively correlated with the expression of vascular endothelial cell marker CD31 and microangiogenesis, confirming that ASRPS inhibited tumor angiogenesis [184].

**Fig. 3:**
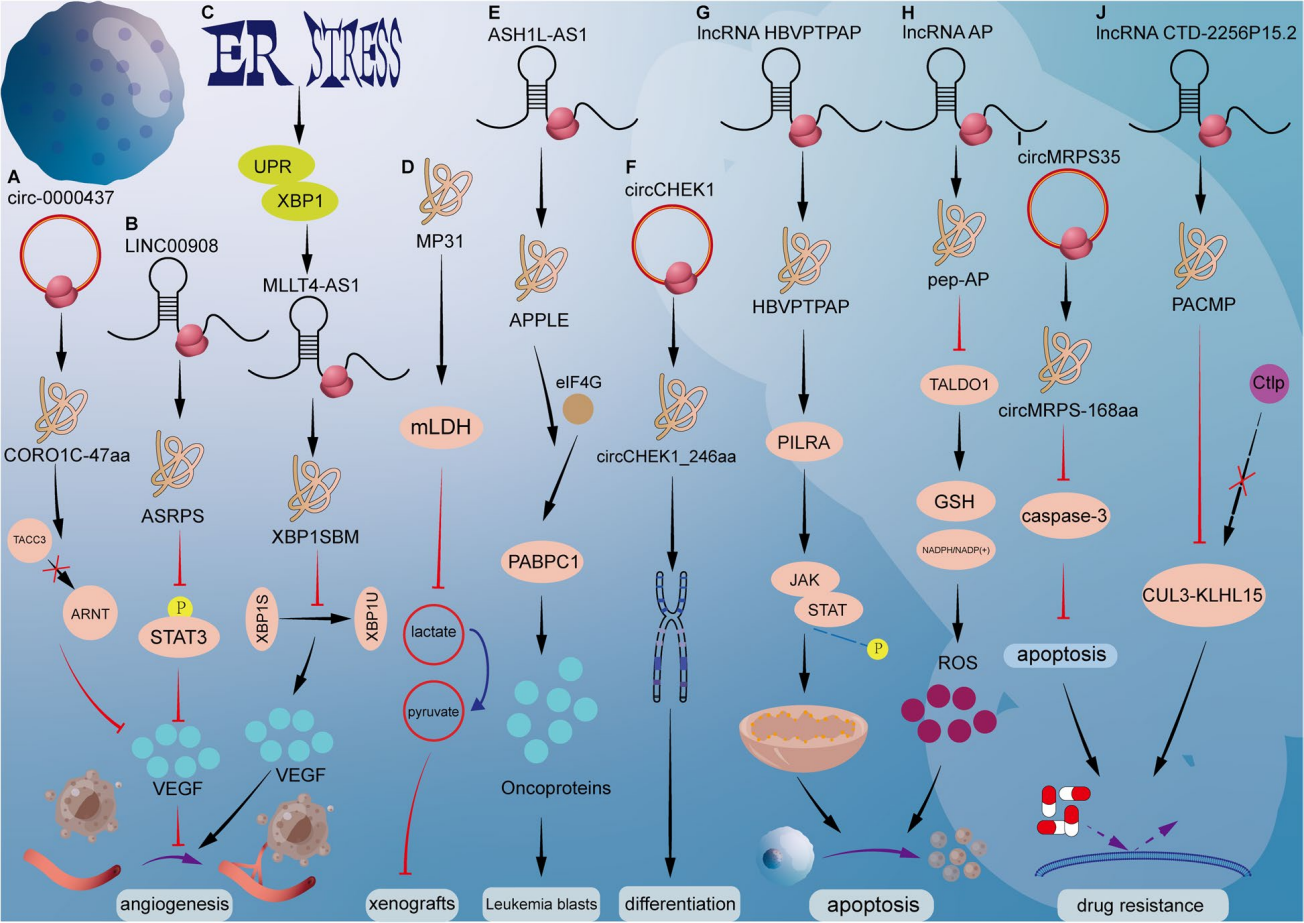
Mechanism of action of peptides in other characteristic phenotypic processes of tumors. **A** CORO1C-47aa via blocking the association between ARNT and TACC3 and then reduces the expression of VEGFA. **B** ASRPS directly bound to STAT3 and down-regulated STAT3 phosphorylation, which led to reduced expression of VEGF. **C** Gln starvation induces ER stress and UPR to activate XBP1 pathway, then XBP1s upregulates transcription of MLLT4-AS1 and expression of XBP1SBM, inhibiting the interaction of XBP1u and XBP1s to enhance the nuclear localization of XBP1s, thereby promoting the transcription and expression of VEGF, and finally drives the metastasis of TNBC. **D** MP31 limits lactate-pyruvate conversion in mitochondria by competing with mitochondrial lactate dehydrogenase (mLDH) for nicotinamide adenine dinucleotide (NAD +) and then inhibited glioblastoma xenografts. **E** APPLE promotes PABPC1-eIF4G interaction and facilitates mRNA circularization and eIF4F initiation complex assembly to support a specific pro-cancer translation program. **F** circCHEK1_ 246aa increased MM CIN and osteoclast differentiation. **G** Interaction between the HBVPTPAP and the PILRA endo-domain activated by the negative regulation of the downstream JAK/STAT pathway, which initiated the mitochondrial pathway to induce apoptosis. **H** pep-AP inhibits PPP, increasing the accumulation of ROS and mitochondrial dysfunction. **I** circMRPS35-168aa suppressed the cisplatin-induced apoptosis via inhibiting the cleavage of caspase-3. **J** PACMP not only prevents CtIP from ubiquitination by inhibiting the CtIP-KLHL15 association but also directly binds DNA damage-induced poly (ADP-ribose) chains

Researchers have also found peptides that promote tumor angiogenesis. Gln-hungry TNBC cells up-regulated XBP1s by activating the UPR/IRE1α-XBP1s pathway, thereby promoting MLLT4-AS1 transcription and XBP1SBM expression, and then XBP1SBM interacted with XBP1s in trans. The activation domain (TAD) blocks the interaction between XBP1u and XBP1s, inhibits the nuclear output of XBP1s, and thus regulates the transcription of VEGF [185] (Fig. 3).

### Other functions and mechanisms of peptides

The peptides encoded by ncRNA have several other functions in addition to those described above. Signaling pathways are critical in cancer research, and researchers have found that HBVPTPAP, a peptide encoded by the lncRNA HBVPTPAP, plays an important role in the JAK/STAT pathway. Further studies showed that when the HBVPTPAP gene was overexpressed, apoptosis-related protein Bax was up-regulated, Bcl2 was down-regulated, cytochrome C expression was increased, and p-STAT3 expression was decreased. Moreover, it was confirmed that HBVPTPAP interacts with PILRA intracellular domain to activate JAK/STAT pathway and initiate mitochondrial pathway to induce apoptosis [186]. Oxaliplatin is used as a tumor chemotherapy drug, and drug resistance will inevitably occur with the extension of the use time. In the process of drug resistance study on oxaliplatin, researchers found that lncRNA AP encoded 37-aa peptide, namely PEP-AP. Experiments have confirmed that pep-AP can inhibit the pentose phosphate pathway (PPP) by inhibiting the expression of TALDO1 protein, increasing ROS accumulation and mitochondrial dysfunction, and finally inducing apoptosis and sensitization to CRC [187]. MP31 is an upstream phosphatase peptide encoded by ORF and tensin congeners (PTEN). It restricts lactate-pyruvate conversion in mitochondria by competing with mitochondrial lactate dehydrogenase (mLDH) for nicotinamide adenine dinucleotide (NAD +). Conditional knockout of MP31 homologues in mouse astrocytes induces glioma formation, shortens overall animal survival, and establishes the tumor suppressive effect of MP31 [188]. The oncogenic peptide APPLE is encoded by noncoding RNA transcripts in acute myeloid leukemia (AML). The peptide is rich in ribosomes, can regulate the translation initiation step to enhance translation, and can maintain a high translation rate for oncoprotein synthesis. APPLE promotes PABPC1-eIF4G interaction and promotes mRNA circularization and eIF4F initiation complex assembly to support specific cancer-promoting translational programs [189]. circCHEK1_246aa interacts with CEP170, which induces MM Chromosomal instability (CIN) and Peripheral blood mononuclear cells (PBMCs) by upregulation of NFATc1 expression, and finally induces MM cell proliferation, drug resistance, and bone disease formation [190]. Tumor drug resistance has always been a key problem in clinical chemotherapy. Studies have found that peptides encoded by ncRNA can promote the development of drug resistance in tumor cells. For example, circMRPS35 can inhibit cisplatin-induced apoptosis by encoding circMRPS35-168aa to inhibit cleavage of caspase-3 [191], Following DNA damage, PACMP binds PAR to promote PARP1-dependent PAR acylation through its electrostatic stabilization of the PAR-acylated PARP1-DNA complex during PAR elongation. Targeting PACMP can inhibit tumor growth, and confer sensitivity to PARP/ATR/CDK4/6 inhibitors, ionizing radiation, epirubicin, and camptothecin. Finally, inhibition of peptides action could be used to enhance existing anticancer therapeutic strategies [192] (Fig. 3).

### Application prospect

The recent surge in life sciences research has cast a spotlight on the translatable noncoding RNAs (ncRNAs) and their profound implications in oncology. This text encapsulates the fundamental translation mechanisms employed by these ncRNAs to encode peptides/proteins and underscores their regulatory influence on cancer dynamics. These peptides/proteins have demonstrated potent functionalities both in the physiological milieu and in controlled laboratory conditions. However, our understanding of these ncRNA-derived peptides/proteins is still in its infancy, with a plethora of them awaiting discovery. The landscape of technologies anchored on translational mechanisms is in constant flux, signaling an ongoing evolution. Concurrently, the real-world clinical implications of these peptides in tumor management warrant further exploration. Over the recent years, the advent of a plethora of anticancer therapeutics, including pathway-specific small molecule inhibitors, antiangiogenic agents, ubiquitin–proteasome inhibitors, monoclonal antibodies, and gene therapies, has reshaped the therapeutic realm. Considering the versatility of the aforementioned peptides/proteins in modulating a broad range of pathways, they hold substantial promise as potential therapeutic targets in cancer treatment.

Distinct peptides have been discovered to exert antitumor effects by impeding the vascularization of cancer cells, consequently obstructing their proliferation rather than triggering direct cellular apoptosis. These peptides can effectively inhibit the signaling of vascular endothelial growth factor (VEGF), a fundamental process that incites new blood vessel formation within tumors. The peptides can curb tumor growth and metastasis via VEGF inhibition while sparing normal cells with low neovascularization demands. Another intriguing approach in cancer therapeutics involves leveraging peptides to incite a tumor-specific immune response. This strategy was recently illustrated in a study that linked a cell-penetrating peptide, termed cytosol localizing internalization peptide 6 (CLIP6), with a model antigen, ovalbumin (OVA) [193]. Unique to CLIP6 is its ability to translocate directly through cell membranes, bypassing the common endocytosis pathway often resulting in endosomal entrapment. In the study, researchers found that the CLIP6-OVA complex facilitated efficient cellular entry and enhanced antigen uptake by antigen-presenting cells, particularly dendritic cells. Furthermore, when administered in vivo with an immune adjuvant, CpG, the CLIP6-OVA complex prompted a potent antigen-specific immune response in mice. Employing the B16/OVA mouse model, a melanoma cancer model that expresses OVA on its cell surface, the researchers assessed the effectiveness of CLIP6-OVA/CpG immunization. The study revealed that two out of six mice receiving this novel immunization regimen became tumor-free, indicating its potential therapeutic efficacy [194].

Cancer vaccines have increasingly garnered attention due to their ability to generate long-lasting immune memory, offering durable anti-tumor effects. Clinically utilized cancer vaccines include Melacine for melanoma and Cima Vax EGF for lung cancer [195–197]. A promising focus for immunotherapy is tumor-specific antigens (TSAs), 90% of which have been identified in human primary tumors and originate from the translation of noncoding regions. Moreover, most of these TSAs derived from nonmutated yet aberrantly expressed transcripts. The efficacy of TSA-based vaccination, as indicated by studies in mice (the strength of antitumor responses after TSA vaccination was influenced by TSA expression and the frequency of TSA-responsive T cells in the preimmune repertoire), demonstrates that individual TSA immunization provides varied degrees of protection against EL4 cells—a protection that is enduring. Consequently, TSAs derived from noncoding regions represent a promising avenue in cancer immunotherapy [198]. Ongoing research continues to uncover a growing number of proteins/peptides encoded by noncoding RNAs (ncRNAs). We anticipate these findings to provide a significant impetus for further clinical exploration, potentially translating into innovative and effective therapeutic strategies.

## Conclusions and future perspectives

In a swirl of scientific fascination, ncRNA-encoded proteins have captured considerable attention. Research has substantiated the existence and underscored the significance of ncRNA-encoded functional peptides. Nonetheless, gauging ncRNA coding potential presents a formidable challenge [199]. Databases employed for predicting interspecies conservation of ORFs, IRES, and m6A in ncRNAs remain incomplete, while experimental validation procedures are still in their infancy [39]. A vast majority of circRNAs stem from protein-encoded exons, potentially overlapping with related mRNAs and complicating the discernment of translation product origins. High-throughput analysis and detection techniques, such as ribosome profiling, face technical hurdles [200, 201]. Pinpointing small peptides necessitates specialized biochemical and bioinformatics approaches infrequently utilized in genome-wide characterization. Furthermore, cell- and tissue-specific expressions add complexity to these assays. Consequently, the true count of translatable sORFs and their biological roles remain enshrouded in mystery.

In this analysis, we delved into recent breakthroughs concerning ncRNA-encoded diminutive peptides governing human cancer behavior. This exploration furnished fresh insights into ncRNA functions and mechanisms. As a result, it prompts the prospect of conducting more in-depth research on ncRNA in several domains, such as the existence of additional functional peptides or proteins encoded by ncRNA; whether past ncRNA studies scrutinized RNA or assessed potential coding functions; the mechanism driving the dynamic translation of ncRNAs encoding functional peptides; whether ncRNAs encoding small peptides undergo posttranslational modification akin to mRNA; and the factors and conditions influencing ncRNA translation. As we peer into the future, functional peptides encoded by ncRNAs could become commonplace in cancer research, therapy, diagnostics, and prognostics, given their developmental potential and clinical applicability. NcRNAs can encode cancer-suppressive peptides/proteins that could be used alongside conventional anti-cancer drugs, or in tandem with traditional radiotherapy and chemotherapy, to bolster the efficacy of cancer treatments. These functionally encoded peptides, entwined with tumorigenesis, present as promising new drug development targets. Researchers are exploring the restoration or enhancement of tumor suppressor peptide/protein functions using vaccinations with synthetic peptides or viral vector vaccines encoding relevant peptide sequences for cancer therapy [202]. The therapeutic potential of these cryptic peptides/proteins, encoded by ncRNAs, is increasingly evident. Furthermore, ncRNA itself can execute biological functions, serving as a molecular marker or potential target. Both functional peptides and ncRNAs can be employed as cancer biomarkers for clinical applications at dual levels of transcription and translation, augmenting the accuracy and specificity of diagnosis and treatment. At this stage, research on ncRNA-encoded peptides is more focused on ncRNAs, and there is still not much research on the peptides themselves. We suggest that research on peptides can be more diversified and more focused on the functions of the peptides, which will allow for a more comprehensive study of the functions of the peptides. For example, CIP2A-BP was introduced into C57BL/6 mice, and then mated with MMTV-PyMT mice to produce new mice that could reduce lung metastasis. A series of in vivo experiments on peptides in animals were carried out by the experimenters, which directly explored that peptides could inhibit the metastasis of breast cancer in vivo experiments, and enriched the content and results of the study. Therefore, we suggest that more diversified peptides experiments can be conducted. Moving forward, the differential expression and prognostic correlation of these peptides/proteins in cancer may be ascertained via further experimental analyses and clinical examinations, such as immunohistochemical analysis of tumor tissue paraffin sections and body fluid assessments.

In this discourse, we explored the notion that genetic information could be transferred from ncRNAs to proteins, potentially playing a significant role in the modulation of specific biological and oncological processes. This insight may aid in elucidating the underpinnings of biological mechanisms and patterns. Given that the realm of functional peptides encoded by ncRNAs remains a relatively nascent field of experimentation and research, the intricacies of its mechanisms, functions, regulatory factors, and prospective clinical and scientific applications warrant and demand continued investigation.

## Data Availability

All relevant data are within the manuscript and its additional files.

## Abbreviations

ncRNA: Noncoding RNA
sORFs: Small open reading frames
Ribo-seq: Ribosome sequencing
IRES: Internal ribosome entry sites
m6A: N6-methylladenosine
CDSs: Coding DNA sequences
miRNA: MicroRNA
CO-IP: Co-Immunoprecipitation
ER: Endoplasmic reticulum
BRI3BP: BRI3-binding protein
GC: Gastric cancer
BC: Breast cancer
AQP2: Aquaporin 2
SEPT2: Septin 2
HNSCC: Head and neck squamous cell carcinoma
ITGB4: Integrin Beta 4
LC-MS: Liquid chromatography-tandem mass spectrometry
MS: Mass spectrometry
HUVEC: Human Umbilical Vein Endothelial Cells
PPP: Pentose phosphate pathway
CIN: Chromosomal instability
HCC: Hepatocellular carcinoma
MM: Multiple myeloma
TNBC: Triple-negative breast cancer
MPM: Micropeptide in Mitochondria
STAT3: Signal transducers and activators of transduction3
PACMP: PAR-amplifying and CtIP-maintaining micropeptide
AML: Acute myeloid leukemia
